# What Drives Task Performance in Fluency Tasks in People With HIV?

**DOI:** 10.3389/fpsyg.2021.721588

**Published:** 2021-10-13

**Authors:** Adrià Rofes, Bárbara Sampedro, Lorena Abusamra, Paola Cañataro, Roel Jonkers, Valeria Abusamra

**Affiliations:** ^1^Center for Language and Cognition Groningen (CLCG), University of Groningen, Groningen, Netherlands; ^2^Linguistics School, University of Buenos Aires, Buenos Aires, Argentina; ^3^Hospital Dr. Diego Thompson, Buenos Aires, Argentina; ^4^Argentina’s National Council for Scientific and Technical Research (CONICET), Buenos Aires, Argentina; ^5^Hospital Interzonal General de Agudos Eva Perón, Buenos Aires, Argentina

**Keywords:** fluency, category, animal, letter, unconstrained, word properties, executive functions

## Abstract

**Introduction:** Fluency tasks require language (i.e., semantics, phonological output lexicon, and phonological assembly) and executive functions (i.e., inhibition; mental set shifting; updating, and monitoring). Little is known about whether people with human immunodeficiency virus (HIV) are more impaired on a specific type of fluency task and what aspects of language and executive functions drive such performance.

**Aims:** To understand (1) whether people with HIV are more impaired in animal, letter, or unconstrained fluency relative to a normative sample; (2) whether there exist differences between tasks relative to the total number of words; and (3) which aspects of executive function and language are involved in their performance.

**Methods:** Data from animal, letter, and unconstrained fluency of 50 Spanish-speaking people with HIV were analyzed. The number of switches and mean cluster size for each task and 10 word properties (e.g., frequency, age of acquisition, length in graphemes) for each of the correct words were measured. A chi-square test was used to address Aim 1, linear mixed effects models for Aim 2, and random forests and conditional inference trees for Aim 3. The results were cross-validated with a normative sample.

**Results:** People with HIV were not more impaired in animal, letter, or unconstrained fluency relative to a normative sample. People with HIV produced fewer words in letter fluency compared to animal and unconstrained fluency. In addition, they produced fewer words in animal fluency compared to unconstrained fluency. Number of switches emerged as the most important variable to predict the total number of correct words when considering the three tasks together and for each task separately. Word frequency was relevant to predict animal fluency, age of acquisition to predict letter fluency, and cluster size to predict unconstrained fluency. These results were cross-validated with the exception cluster size.

**Conclusion:** People with HIV rely on language (phonological output lexicon, not necessarily semantics) and executive functioning (updating and monitoring) to produce words in fluency tasks. These results concur with the current literature. Future work may correlate fluency scores with other tests measuring language and executive functions or study other types of fluency tasks (e.g., action, cities, supermarket, and professions).

## Introduction

Fluency tasks are widely used to assess people with neurological disorders, including people with the human immunodeficiency virus (HIV) (e.g., Hestad et al., 1993; Becker et al., 1997; White et al., 1997; Millikin et al., 2004; Woods et al., 2004; Abusamra et al., 2012; Vonk et al., 2020; Rofes et al., 2021; see cf. Damos et al., 1997). Typically, clinicians and researchers give participants 60s to produce as many words as possible starting with a specific letter of the alphabet (letter fluency, for example, “F,” “A,” and “S”) or words that belong to a specific semantic category (category fluency, e.g., “animals,” “fruits,” and “vegetables”). They can also ask participants to produce as many words as possible belonging to any word category (unconstrained fluency, e.g., Beausoleil et al., 2003; Gonçalves et al., 2017). In this paper, we studied the strategies that people with HIV use to produce words in letter fluency, category fluency (specifically, animal fluency), and unconstrained fluency.

People with HIV tend to be more impaired in letter fluency than in category fluency (Hestad et al., 1993; Becker et al., 1997; White et al., 1997; Millikin et al., 2004; Woods et al., 2004; see cf. Damos et al., 1997). Impairments in unconstrained fluency have been reported to a similar extent as impairments in category fluency, albeit we are only aware of one report using unconstrained fluency in people with HIV (Abusamra et al., 2012). The overall pattern of performance of these individuals in fluency tasks has been attributed to difficulties with lexical access, lexical retrieval, and executive functions (i.e., rule-guided search strategies), relative to subcortical brain damage in frontal-basal ganglia circuits (e.g., Aylward et al., 1993; White et al., 1997; Millikin et al., 2004; Woods et al., 2004). These difficulties have also been used to explain why people with HIV have difficulties finding words during spontaneous speech, while object naming tends to be spared (McCabe et al., 2002, 2008; Sheppard et al., 2017).

As of today, a clear picture of what aspects of language (i.e., semantics, phonological output lexicon, and phonological assembly) and executive functions (i.e., mental set shifting, information updating and monitoring, and inhibition of possible responses) explain the total number of words in fluency tasks in people with HIV and how these factors interact with each other is not yet available. Shedding light onto this issue, some authors have indicated that people with HIV not only produce fewer words than people without brain damage in fluency tasks, but also fewer switches (e.g., in an animal fluency task, starting with names of pets, and then “changing” to names of farm animals), more word repetitions, and more words of a wrong category (e.g., Millikin et al., 2004; Woods et al., 2004; Abusamra et al., 2012).

In the current article, we go beyond the classic method of counting the total number of words, by considering variables that can be extracted from the fluency tasks themselves. A data-driven effort to use this approach in a relatively large number of word properties, as well as switches and clusters, was recently reported (Rofes et al., 2020). In that study, the authors looked at nine word properties and numbers of clusters and switches in the responses to an animal fluency task of English speakers with probable Alzheimer’s disease (AD). Their results indicated that the total number of correct words in animal fluency could be predicted by age of acquisition and number of switches. Finding that these two factors were relevant, and not others, was used to argue that the difficulties that people with AD have in animal fluency are due to issues at the lexical level, not necessarily the semantic level, and in executive functions, particularly, in information updating and monitoring.

In our current study, we used a similar approach to that of Rofes et al. (2020) with the data of Spanish speakers with HIV. The idea behind studying clusters, switches, and word properties relates to the need to understand how well a participant performs in any neuropsychological task and how this performance is achieved (e.g., Kaplan, 1990; Abwender et al., 2001). To achieve this goal, looking only at the total number of words that people produce or at the differences between types of fluency tasks may not be sufficient. This is because, to perform any type of fluency task, language and executive functions are needed, albeit to potentially different degrees, and because the impairments that people with neurological disorders have may affect both language and executive functions (e.g., Henry et al., 2004; Shao et al., 2014; Whiteside et al., 2016; Rofes et al., 2020). To further motivate this approach, below we describe the study of clusters, switches, and word properties of fluency tasks:

### Studying Clusters and Switches in Fluency Tasks

Clusters in fluency tasks refer to groupings of successively generated words that belong to the same semantic family (e.g., Raskin et al., 1992; Troyer et al., 1997, 1998a,b; Troyer, 2000). In letter fluency, examples of clusters include words starting with the first two same letters (e.g., *pala*/shovel, *papa*/potato, and *pantalla*/screen). In some cases, these two letters coincide in their syllabic structure (e.g., *pala*/shovel and *papa*/potato), and in others, only the letters coincide (e.g., *papa*/potato and *pantalla*/screen). There are also clusters in which the words differ by one or two vowels (e.g., *pelo*/hair, *palo*/stick; *pelota*/ball, and *paleta*/paddle). These clusters are usually short (two or three elements per cluster). Other criteria, such as words that rhyme or words that are homonyms, are not very productive in Spanish. In unconstrained fluency, the semantic categories used are many and diverse. Frequent clusters include clothing (e.g., shirt, tie, pants, shoe, and T-shirt), parts of the body (e.g., hands, feet, legs, hair, and eyes), transportation means (e.g., car, train, bus, and airplane), colors (e.g., white, black, red, blue, yellow, green, and purple), parts of the house (e.g., bathroom, living room, kitchen, and dining room), furniture (e.g., table, chair, closet, and armchair), plants and flowers (e.g., azalea, gladiolus, and petunia), and library items (e.g., pencil, paper, rubber, and scissors). In animal fluency, examples of clusters include living environment (e.g., African animals), taxonomy (e.g., bovine, feline), and human use (e.g., farm animals). For example, the sequence “cow, sheep, donkey, horse, lion, tiger” contains two clusters: farm animals (i.e., “cow” to “horse”) and felines (i.e., “lion” and “tiger”). For each cluster, it is also possible to obtain its size. Cluster size is calculated based on the number of words within the cluster minus one. Hence, individual words cannot form clusters. In the example above, the cluster sizes are 3 and 1, respectively. People with temporal lobe lesions, individuals with Parkinson’s disease, and people with Alzheimer’s disease (AD) produce clusters of smaller size than healthy individuals. These difficulties have been related to impairments in semantic memory (Troyer et al., 1998a,b). Cluster size has been shown to be a relevant variable in unconstrained fluency (e.g., Beausoleil et al., 2003; Gonçalves et al., 2017). This is because unconstrained fluency does not have any *a priori* rules which participants need to follow to produce the words, as opposed to letter fluency and animal fluency. Therefore, participants are able to produce words in larger groups than in other types of fluency tasks.

Switches are instances in which participants change the uttering of items of one subcategory to another subcategory (e.g., Troyer et al., 1997; Abwender et al., 2001). In the example above (i.e., “cow, sheep, donkey, horse, lion, and tiger”), the participant produces one switch when the cluster of farm animals is replaced by a new cluster containing felines. Authors argue that switches reflect two aspects of executive functions, namely, information updating and monitoring (Miyake et al., 2000; Rofes et al., 2020). This is because the change between subcategories requires renewing the criteria used to search for words, keeping track of the words that were already produced, and continuously adhere to the task instructions (e.g., produce only animals), all within the same task. Other authors have also indicated that switches may also reflect lexical-semantic abilities, as people that produce more switches also need to produce further exemplars of different subcategories (Mayr, 2002).

### Studying Word Properties in Fluency Tasks

Word properties (or psycholinguistic variables) are characteristics of words that can be extracted using corpora (e.g., frequency ratings as found in collections of books, subtitles, and spoken language), running questionnaires to healthy individuals (e.g., how familiar they are with the word; how concrete is the word; when they think they learned the word), and by measuring physical characteristics of the words (e.g., number of graphemes or phonemes). The study of word properties of language tasks is relevant because it may indicate impairments at specific language levels (e.g., Whitworth et al., 2014; Rofes et al., 2019; Alyahya et al., 2020; Bemani et al., 2021).

Difficulties relative to word properties such as concreteness, imageability, familiarity, valence, and arousal relate to impairments of the semantic level, the place where meanings are stored and operationalized to understand concepts and ideas (e.g., Nickels and Howard, 1994, 1995; Guasch et al., 2016). Damage to the semantic level can lead to difficulties producing and comprehending language in the spoken and written form (Whitworth et al., 2014). Concreteness is obtained by asking people the degree to which a concept refers to an entity that can be perceived (e.g., “table” rates high in concreteness, while “virtue” rates low; Paivio et al., 1968). Imageability indicates the degree to which a concept evokes a mental image or sensory experience (e.g., “house” is high in imageability, while “hope” is low; Paivio et al., 1968). Familiarity measures indicate how often individuals are in contact with or use certain words (e.g., “dog” would be high in familiarity, while “cosine” would be low; Noble, 1953). Valence reflects the degree to which a concept is pleasant. It can be measured by asking individuals to rate words on a scale ranging from “happy” to “sad” (e.g., “smile” would be high in valence, while “cancer” would be low; Guasch et al., 2016). Finally, arousal indicates the degree of activation of a concept. It is obtained by asking individuals to rate words on a scale ranging from “energized” to “calm” (e.g., “attack” would be high in arousal, while “nap” would be low; Guasch et al., 2016).

Age of acquisition, frequency, orthographic, and phonological neighborhood (or similarity) relate to impairments in the phonological and orthographic output lexica, which are stores of spoken or written word forms (e.g., Gilhooly and Watson, 1981; Cuetos et al., 2010; Whitworth et al., 2014). Difficulties with these word properties can indicate damage to the output lexica or access to the lexica from the semantic level. Damage to the phonological output lexicon and the orthographic output lexicon can dissociate. Therefore, an individual can have difficulties in oral naming and speaking, but not in written naming or written description. Age of acquisition ratings is obtained by asking people to indicate when they learned word in the written or spoken form (e.g., “airplane” is learned early in life and therefore people give this word a low score for age of acquisition; while a word like “anvil” is learned later in life, and therefore, people give it a higher score for age of acquisition; Carroll and White, 1973). Frequency indicates how many items a word appears in a large collection of written and/or spoken corpora (e.g., “mother” occurs more frequently than “pharaoh”; Kučera and Francis, 1967). Orthographic and phonological neighborhood are independent measures of lexical similarity. They are obtained by counting the number of words that can be created by substituting one letter/phoneme of the target word, given a corpus (e.g., “soul” rates high in neighborhood, because we can obtain words that are pronounced similarly, such as “bowl,” “coal,” and “dole”). On the contrary, “mountain” has only one phonologically similar neighbor, “fountain” (Duchon et al., 2013).

Finally, length in graphemes reflects issues in phonological encoding, a language level where phoneme strings are put together in preparation to be converted into motor commands (e.g., Shallice et al., 2000; Nickels and Howard, 2004). This measure is obtained by counting the number of graphemes in a word (e.g., “dog” has three graphemes, while “velociraptor” has 12 graphemes).

### Aims and Predictions

This is a data-driven study to understand (1) whether people with HIV are more impaired in animal, letter, or unconstrained fluency relative to a normative sample; (2) whether there exist differences between tasks relative to the total number of words; and (3) what factors (i.e., linguistic and executive) influence their performance in fluency tasks, as measured by the total number of words.

Based on previous reports, we expect: (a) people with HIV to be more impaired (i.e., to produce significantly fewer correct words relative to a group of 30 individuals without HIV matched for age and education, given the normative sample of Ferreres et al., 2007) in letter fluency, relative to animal and unconstrained fluency (e.g., White et al., 1997; Millikin et al., 2004); (b) people with HIV to produce fewer correct words (i.e., regardless of whether or not they perform below the norm) on letter fluency, relative to animal and unconstrained fluency (e.g., Vonk et al., 2020). For the comparison between animal and unconstrained fluency, we expect no differences; (c) total number of correct words in animal fluency and unconstrained fluency to be better explained by word properties that are typically related to lexical-semantic processes (i.e., age of acquisition, concreteness, familiarity, frequency, imageability, arousal, valence). Also, we expect variables that are typically related to the (phonological/orthographic) output lexica and buffer to explain the total number of words in letter fluency (i.e., age of acquisition, frequency, length in graphemes, and orthographic/phonologic neighborhood) (e.g., Whitworth et al., 2014; Rofes et al., 2019; Alyahya et al., 2020; Bemani et al., 2021). We expect cluster size and number of switches to be relevant to explain fluency tasks. Cluster size may be particularly relevant for unconstrained fluency. However, given the scarcity of studies (e.g., Beausoleil et al., 2003; Gonçalves et al., 2017; Rofes et al., 2020), it is hard to predict whether these factors may be more relevant to explain total number of words in any of the tasks or whether they may be more relevant than word properties.

## Materials and Methods

### Participants

Fifty Spanish-speaking people with HIV (i.e., HIV-1 positive) participated in this study. All participants were from Buenos Aires, Argentina. They were 35 males, 15 females of mean age 40 (SD=9), and mean education 13years (SD=4). All participants were screened for the following inclusion criteria: ≥18years of age; native Spanish speaker; no developmental language problems, including reading and writing; no history of neurological or psychiatric disease; normal scores in the Mini mental state Examination (≥27/30, Butman et al., 2001); normal scores in two of the following four tests (Trail Making test; Strauss et al., 2006; Stroop Test, Golden, 1994; Digit Span backward/forward, WMS-R; Weschler, 1974; Hayling Test, Abusamra et al., 2007); currently working or able to work. In Table 1, we included a summary of the cognitive scores.

**Table 1:** Scores cognitive tests.

Test	Mean z-score (SD)
Trail making test (Strauss et al., 2006)	
-A	1.13 (1.39)
-B	1.71 (2.18)
Stroop test (Golden, 1994)	
-Color	−0.73 (0.94)
-Word	−1.24 (0.86)
-Color-word	−0.71 (0.99)
-Resistance interference	−0.16 (0.99)
Digit span (WMS-R; Weschler, 1974)	
-Forward	1.27 (4.18)
-Backward	−1.34 (5.26)
Hayling test (Abusamra et al., 2007)	
-A (time)	0.25 (1.14)
-B (time)	−0.05 (1.12)

Of the 50 patients evaluated, 36 people were on antiretroviral treatment. The mean viral load of the 10 patients who were not under treatment was 34,201 (log10: 4,53). In addition, seven of them had a T lymphocyte count/CD4>350. The mean viral load of the remaining four patients not receiving treatment was not accessible. Before entering the research, participants were filled in on the details of the project and asked to sign an informed consent form approved by the Ethics Committee of the Hospital Interzonal General de Agudos Eva Perón. The consent explained what the battery consisted of, the number of sessions (3 max.), and the maximum time per session (45min).

### Fluency Tasks, Scoring, and Reliability

We administered three fluency tasks to each participant. In each task, participants were given 120s to say as many words as possible belonging to the category animals [i.e., animal fluency, e.g., *perro*, *gato*, *mono*, and *elefante* (dog, cat, monkey, and elephant)]; starting with the letter “p” [i.e., letter fluency, e.g., *papá*, *perchero*, *planta*, and *pelota* (father, hanger, plant, and ball); or “any type of word, without forming a sentence” [i.e., unconstrained fluency, e.g., *bienestar*, *flor*, *nube*, and *computadora* (wellness, flower, cloud, and computer)]. Similar to other studies, participants were told to close their eyes. Also, in unconstrained fluency participants were also told not to produce proper nouns (e.g., Juan) or numbers (e.g., seven). The latter two criteria were not specified in animal or letter fluency tasks.

Participants were given 120s (vs. 60s) to respond to each fluency task. The use of 120s has been recommended to obtain more information regarding lexico-semantic search processes. This argument has been particularly stressed for unconstrained fluency, upon consideration that in the first 30s participants mostly produce items with a high degree of prototypicality (i.e., a word property reflecting that can be related to semantic processing, e.g., “robin” or “dove” a more typical concepts of the category “bird” than “ostrich” or “penguin”), and only after 30s participants show further effort to search for words, as shown by the use of different word subcategories (e.g., Beausoleil et al., 2003).

We followed the criteria by Troyer (2000) to score the tasks for the mean cluster size and the number of switches. In unconstrained and animal fluency, a cluster consists of at least two successively generated words that belong to the same (sub)semantic category. In letter fluency, the cluster does not imply a semantic category but rather words that begin with the same two letters, that are differentiated by a vowel sound/rhyme or are homonymous. Since a cluster must contain more than one element, the cluster size is defined as the number of elements in a category minus one. The mean of the cluster size was calculated by the sum of the size of each cluster and its division by the number of clusters. The number of switches was calculated by counting the transitions from one cluster to another, so the number of switches is equal to the number of clusters minus one.

Ten word properties were extracted for each word counting toward the total score (i.e., disregarding repetitions, proper nouns, and any other criteria outlined above). The word properties were as follows: age of acquisition, arousal, concreteness, familiarity, frequency, imageability, length in graphemes, orthographic neighborhood, phonologic neighborhood, and valence. To minimize the number of instances where words did not have a value for a word property (i.e., empty values), we turned all the words our participants said to singular and masculine. Also, we excluded diminutives. This is because words, such as “gata” (female cat) or “amigos” (male friends), may not have values for many of the word properties that were asked in a subjective way. However, databases most typically include ratings for “gato” (male cat) and “amigo” (male friend). Note that for word frequency and length in phonemes, we used the words as produced by our participants.

The database of Duchon et al. (2013) was used to retrieve values for frequency (i.e., log based on a corpus of subtitles of movies and TV shows), orthographic and phonologic neighborhood (number of written/spoken substitution neighbors using Latin American phonology), familiarity (how familiar are you with the word?), imageability (how much does the word relate to a sensory experience?), and concreteness (how concrete is the word?). The last three word properties were obtained by asking university students to rate the words in a 7-point Likert scale (Duchon et al., 2013). We used the database of Guasch et al. (2016) to fill in values for words that were not included in Duchon et al. (2013). The database of Guasch et al. (2016) was used to retrieve values for valence (extent to which an emotion is pleasant or unpleasant, from sad to completely happy) and arousal (degree of activation of a word, from calm to energized). These two word properties were obtained by asking university students to rate the words in a 9-point Likert scale (Guasch et al., 2016). Alonso et al. (2015) were used to retrieve values for age of acquisition. This database was obtained by asking university students to rate words using an 11-point Likert scale. Finally, length in graphemes was obtained by counting the graphemes in the words.

### Analyses

Analyses were conducted in R (R Core Team, 2017). To assess whether people with HIV are more impaired in animal, letter, or unconstrained fluency relative to a normative sample, we ran a chi-square test [i.e., chisq.test(dataframe)] comparing the number of people with HIV who produced scores within and below the norm (given the total number of correct words) in each of the fluency tasks (i.e., animal, letter, and unconstrained) relative to a normative sample (Ferreres et al., 2007). The normative sample we used include data from 180 Spanish-speaking participants from Argentina, divided into six subgroups of 30 people, each defined by aged and education. The subgroups include participants from 30 to 88years of age (divided into three groups: 30–49, 50–64, 65–88). Each of these age groups is further divided into two groups, according to whether the individuals received ≤10years of education or>10years of education. It is important to stress that participants in the normative sample were also given 120s to respond to the same three fluency tasks (i.e., animal, letter, or unconstrained fluency). For our analysis, we had to exclude six participants with HIV because they were younger than 30 and our normative sample only included participants older than 30years of age (i.e., Ferreres et al., 2007).

To assess whether there exist differences between the three fluency tasks relative to the total number of words, we performed a linear mixed effect model including fixed effects for Task and random effects for Participant [i.e., mixedModel <−lmer (TotalWords ~ Task + (1|Participant), dat]. We also computed pairwise comparisons [i.e., pairs (emmeans (mixedModel, “Task”)]. We used the packages lme4 (Bates et al., 2015) and emmeans (Lenth et al., 2018).

To understand what factors (i.e., linguistic and executive) influence performance (as measured by the total number of words) in fluency tasks in people with HIV, we performed random forests/conditional inference trees. The analyses were structured in four consecutive steps: The first three steps were dedicated to running two machine learning algorithms and the fourth step to cross-validate the results with two stochastic measures that are more commonly used in our field.

First, we compared the total word scores of each individual with HIV to the appropriate normative sample using data from our group (Ferreres et al., 2007). This approach was deemed superior to collecting new data, provided the large number of participants in our database. Data for each individual with HIV were compared to the appropriate normative sample with modified t tests (one-tailed), using the computer program Singlims_ES (Crawford et al., 2010). This test allowed us to understand whether the total word scores of each participant were significantly different from those of the normative sample. Hence, we could establish people with HIV who produced a total number of words for all fluency tasks together and for each task individually that was within or below the norm. These values were not possible to calculate for the three tasks together, because in the normative protocol, there is no report of mean values for the three tasks together or results for each individual that was included in the standardization (Ferreres et al., 2007).

Second, to understand the influence of the 10 word properties, mean cluster size, and number of switches on the responses of people with HIV, we ran a machine learning algorithm called random forests. It is desirable to use random forests when the sample size that is entertained is small and when the data contain many predictor variables, some of which may be correlated (Breiman, 2001; Strobl et al., 2008). In our study, the sample size is relatively small (*N*=50). Also, we entered 10 variables in the prediction models, some of which are known to correlate with one another. For example, concreteness with imageability (e.g., Marcel and Patterson, 1978), frequency with age of acquisition (e.g., Carroll and White, 1973; Ellis and Morrison, 1998), frequency with familiarity (e.g., Gernsbacher, 1984), and frequency with length (e.g., Hauk and Pulvermüller, 2004). In a similar way to a recent study from our group (Rofes et al., 2020), we used the statistical program R to run random forests for regression and “variable selection” on our data. That is, to understand which are the best variables to predict the total number of words in fluency tasks, given a specific sample of people with neurological and/or language impairments.

To run random forests, we followed three steps: (1) generating a random forest with unbiased conditional inference trees (Strobl et al., 2008). To do that, we used the cforest function (Hothorn et al., 2017); (2) extracting the relative importance of each prediction using the conditional permutation variable importance, as indicated by the varimp function (Hothorn et al., 2017). Importance reflects how well each variable predicts the dependent variable (i.e., total number of words in the three tasks together; total number of words in each fluency task separately). The removal of a given variable from the model may result in a decrease in model prediction accuracy. When this occurs, that variable is ranked highly in terms of importance (Strobl et al., 2008); (3) estimating predictor accuracy including only informative predictions. To do that, we used leave-one-out cross-validation. This is a procedure in which the classifier is trained on a data set in which one data point (i.e., one participant) is omitted at a time. The value of the observation with the omitted data point is then predicted and saved. This procedure is repeated for each data point. To finalize this step, we examined the relation between the actual values and the predicted values of the total number of words (considering the three tasks together or each task individually). At the end, we evaluated the accuracy of the predictions, as measured by R^2^, root mean squared error (RMSE), and mean absolute error (MAE). More information on this procedure can be found in Tagliamonte and Baayen (2012). Sample R scripts to run these analyses can be found in de Aguiar et al. (2015).

Third, to examine interactions among variables and in addition to random forests, we used another machine learning algorithm, namely, conditional inference trees. This second algorithm identifies points along the scale of a variable where the prediction values of the dependent measure change significantly. These points are called “split points.” The end result of conditional inference trees is a tree-like representation, with nodes representing split points for variables that are significant. In a previous paper, this algorithm indicated that mean age of acquisition scores above 6 (on a scale of 1–10) and a greater number of switches predicted people with AD to produce more words in an animal fluency task (Rofes et al., 2020).

Fourth, we cross-validated the results by comparing the total number of words in the three fluency tasks together and in each of the tasks individually for people with HIV who performed below and within the normal range (based on one-tailed modified t tests, as indicated above). We ran Wilcoxon tests with the independent variable group (below normal vs. within normal) and the dependent variable factor (variable shown to be relevant in the conditional inference trees). Our data were not normally distributed, as indicated by the Shapiro test. Hence, we used non-parametric statistics.

The data we used in this study can be found in the Supplementary Material.

## Results

### Descriptive Statistics

In Table 2, we provided values for the total number of people who produced significantly fewer words in each of the three fluency tasks relative to a normative sample (Ferreres et al., 2007). We also included the mean and standard deviation for the total number of words and each of the linguistic/executive function measures we considered in this study. We provided these values for the three tasks together and for each task separately.

**Table 2:** Descriptive statistics.

	Impaired	Total words	Cluster size	Number switches	Frequency	Imageability	Familiarity	Length graphemes	Orth similarity	Phon similarity	Concreteness	Valence	Arousal	Age of acquisition
Animal	10/50	30 (10)	1.9 (0.5)	6.4 (2.7)	0.8 (0.1)	6.3 (0.1)	5.7 (0.1)	6.3 (0.3)	6.9 (1.6)	16.9 (3.5)	6.1 (0.1)	5.1 (0.8)	4.6 (1.2)	4.3 (0.3)
Letter	4/50	23 (7)	1.6 (1.2)	3.8 (2.5)	0.9 (0.3)	5.7 (0.4)	5.8 (0.2)	6.1 (0.7)	9.0 (3.3)	18.5 (6.5)	5.3 (0.3)	5.3 (0.8)	4.1 (0.7)	4.9 (0.7)
Unconstrained	9/50	45 (16)	2.3 (0.9)	9.5 (4.6)	1.4 (0.3)	6.0 (0.4)	6.1 (0.1)	6.6 (0.7)	7.1 (1.5)	16.3 (3.5)	5.6 (0.4)	5.3 (0.7)	4.6 (0.9)	4.3 (0.5)
All_tasks	NA	98 (28)	2.1 (0.5)	19.6 (7.8)	1.1 (0.2)	6.0 (0.2)	5.9 (0.1)	6.4 (0.4)	7.4 (1.3)	16.9 (2.8)	5.7 (0.2)	5.2 (0.5)	4.4 (0.6)	4.4 (0.3)

Impaired,number of people with HIV (*N*=50) who produced significantly fewer words, relative to a normative sample (Ferreres et al., 2007). NA,number not available because the normative sample (Ferreres et al., 2007) does not consider values for the three tasks together or values for each person included in the standardization.

### Differences in the Number of Impaired People per Task, Relative to Normative Data

The number of people with HIV who produced significantly fewer words relative to a normative sample (Ferreres et al., 2007) was 10 for animal fluency, four for letter fluency, and 11 for unconstrained fluency. A chi-square test indicated no differences in the number of impaired and non-impaired participants across tasks (X^2^[2]=4.2437, *p*=0.12).

### Differences in the Total Number of Correct Words

People with HIV produced significantly more correct words in unconstrained fluency (*m*=45; SD=16) than letter fluency (*m*=23; SD=7; *ß*=−21.2, SE=1.6, *t*=−13.315, *p*=0.001) and animal fluency (*m*=30; SD=10; *ß*=−14.4, SE=1.6, *t*=−9.052, *p*=0.001). Also, they produced more correct words in animal fluency than in letter fluency (*ß*=6.8, SE=1.6, *t*=4.263, *p*=0.001).

#### Factors Influencing Performance

##### All Fluency Tasks

The random forests regression model, computed to select variables ranking high in importance, explained 52% of variance in the dependent measure total number of words (*R*^2^=0.52; RMSE=19.22; MAE=15.21) after leave-one-out cross-validation. Also, the most informative variables in the regression of total number of words were, in order of higher to lower importance: number of switches, frequency, age of acquisition, imageability, familiarity, and mean cluster size.

**Figure 1:**
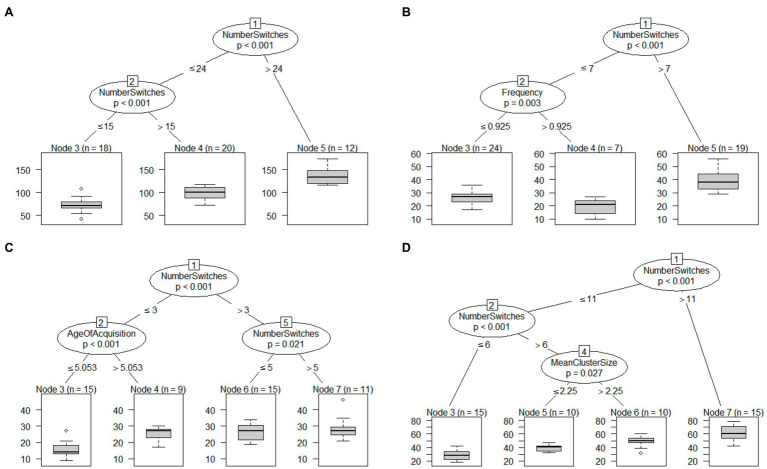
Conditional inference trees for all tasks together **(A)**, animal fluency **(B)**, letter fluency **(C)**, and unconstrained fluency **(D)**.

Conditional inference trees computed by using the variables as shown important in random forests identified no interaction (*R*^2^=0.51; RMSE=17.34; MAE=13.82). However, they indicated two split points for number of switches. The split point at the highest node of the tree (node 1) indicated that participants that produced more than 24 switches (*n*=12), produced a significantly greater number of words compared to participants with number of switches lower or equal than 24 (*n*=38; *X*^2^=38.224, *p*=0.001). Furthermore, within those participants with number of switches lower than or equal to 24 (*n*=38), we found another split point (node 2), whereby people producing more than 15 switches (*n*=20) produced more words than people producing fewer or equal than 15 switches (*n*=18, *X*^2^=20.165, *p*=0.001; see Figure 1A).

##### Animal Fluency

The random forests regression model, computed to select variables ranking high in importance, explained 59% of variance in the dependent measure total number of words (*R*^2^=0.588; RMSE=6.13; MAE=4.49) after leave-one-out cross-validation. Also, the most informative variables in the regression of total number of words were, in order of higher to lower importance: number of switches, age of acquisition, frequency, mean cluster size, familiarity, and arousal.

Conditional inference trees computed by using the variables as shown important in random forests identified an interaction between number of switches and frequency (*R*^2^=0.538; RMSE=6.49; MAE=5.26). The split point at the highest node of the tree (node 1, for number of switches) indicated that participants that produced more than seven switches (*n*=19) and produced a significantly greater number of words compared to participants with the number of switches lower than or equal to seven switches (*n*=31, *X*^2^=33.121, *p*=0.0001). Furthermore, among participants with number of switches lower than or equal to 7, there was a further split. This second split (node 2, for the variable frequency) indicated that participants with mean frequency higher than 0.925 (*n*=7) produced significantly fewer words compared to participants with mean frequency equal or lower than 0.925 (*n*=24, *X*^2^=11.491, *p*=0.003; see Figure 1B).

Wilcoxon tests showed that the 10 people with HIV with scores below the norm in animal fluency produced fewer switches (*m*=4.2; SD=1.9) than the remaining 34 people with HIV with scores within the norm (*m*=7.38; SD=2.5; *W*=56; *p*=0.001). Also, people with scores below the norm in animal fluency produced words of significantly higher frequency (*m*=0.92; SD=0.17) than the remaining 34 people with HIV with scores within the norm (*m*=0.77; SD=0.13; *W*=259; *p*=0.012).

##### Letter Fluency

The random forests regression model, computed to select variables ranking high in importance, explained 30% of variance in the dependent measure total number of words (*R*^2^=0.301; RMSE=6.13; MAE=4.67) after leave-one-out cross-validation. The most informative variables in the regression of total number of words were, in order of higher to lower importance: number of switches, age of acquisition, valence, phonological similarity, and imageability.

Conditional inference trees computed by using the variables as shown important in random forests identified an interaction between number of switches and age of acquisition (*R*^2^=0.11; RMSE=6.89; MAE=5.18). The split point at the highest node of the tree (node 1, for number of switches) indicated that participants that produced more than three switches (n=26) produced a significantly greater number of words compared to participants with number of switches lower or equal than 3 (*n*=24, *X*^2^=11.629, *p*=0.0001). Furthermore, among participants with number of switches lower than or equal to 3, there was a further split. This second split (node 2, for the variable age of acquisition) indicated that participants with mean age of acquisition higher than 5.053 (*n*=9) produced a significantly number of words compared to participants with mean age of acquisition equal or lower than 5.053 (*n*=15, *X*^2^=11.468, *p*=0.0003; see Figure 1C).

Wilcoxon tests showed that the four people with HIV with scores below the norm in letter fluency produced fewer switches (*m*=1.75; SD=0.95) than the remaining 40 people with HIV with scores within the norm (*m*=4.2; SD=2.58; *W*=29; *p*=0.003). Also, people with scores below the norm in letter fluency produced words of significantly lower age of acquisition (*m*=4.02; SD=0.71) than the remaining 40 people with HIV with scores within the norm (*m*=5.03; SD=0.63; *W*=22; *p*=0.014).

##### Unconstrained Fluency

The random forests regression model, computed to select variables ranking high in importance, explained 52% of variance in the dependent measure total number of words (*R*^2^=0.515; RMSE=10.75; MAE=8.73) after leave-one-out cross-validation. Also, the most informative variables in the regression of total number of words were, in order of higher to lower importance: number of switches, frequency, mean cluster size, arousal, and imageability.

Conditional inference trees computed by using the variables as shown important in random forests identified an interaction between number of switches and mean cluster size (*R*^2^=0.618; RMSE=9.54; MAE=8.13). The split point at the highest node of the tree (node 1, for number of switches) indicated that participants that produced more than 11 switches (*n*=15) produced a significantly greater number of words compared to participants with number of switches lower or equal than 11 (*n*=35, *X*^2^=33.495, *p*=0.0001). Furthermore, among participants with number of switches lower than or equal to 11, there was a further split. This second split (node 2, for number of switches) indicated that participants with number of switches higher than 6 (*n*=20) produced a significantly higher number of words compared to participants with the number of switches equal to or lower than 6 (*n*=15, *X*^2^=16.128, *p*=0.0001). Finally, we found another split among participants with the number of switches greater than 6. The third split (node 4, for mean cluster size) indicated that participants with mean cluster size greater than 2.25 (*n*=10) produced more words than participants with mean cluster size lower than or equal to 2.25 (*n*=10, *X*^2^=7.313, *p*=0.0136; see Figure 1D).

Wilcoxon tests showed that the 11 people with HIV with scores below the norm in unconstrained fluency produced fewer switches (*m*=6.54; SD=2.91) than the remaining 33 people with HIV with scores within the norm (*m*=10.75; SD=4.8; *W*=80.5; *p*=0.006). The results for mean cluster size could not be cross-validated, as people with scores below the norm in unconstrained fluency did not produce significantly fewer switches (*m*=1.93; SD=0.63) than the remaining 33 people with HIV with scores within the norm (*m*=2.47; SD=0.99; *W*=123 *p*=0.1154).

## Discussion

This study had three main aims: (1) to understand whether people with HIV are more impaired in animal, letter, or unconstrained fluency relative to a normative sample; (2) to assess whether individuals with HIV produce different numbers of words in the three fluency tasks; and (3) to reveal which aspects of language (i.e., semantics, phonological output lexicon, and phonological assembly) and/or executive functions (i.e., inhibition of possible responses; mental set shifting; information updating, and monitoring) may explain the performance of people with HIV in fluency tasks. To respond to these aims, we used a data-driven approach that works well with single cases, relatively small sample sizes, and with factors that correlate with one another (e.g., concreteness with imageability, Marcel and Patterson, 1978; frequency with age of acquisition, Carroll and White, 1973; Ellis and Morrison, 1998).

Relative to Aim 1, our results indicate that people with HIV were not significantly more impaired in any of the three fluency tasks relative to a normative sample (Ferreres et al., 2007). Finding no differences between animal and unconstrained fluency was within our predictions. However, we expected letter fluency to be more impaired than category fluency, but this was not the case. The latter result speaks against the studies by White et al. (1997) and Millikin et al. (2004). However, in these two studies, the authors included people with HIV that where at a more advanced stage of the disease and, therefore, also cognitively impaired. In our data set, we only included individuals who were not cognitively impaired, as indicated by normal scores in the MMSE (≥27/30, Butman et al., 2001), normal scores in two of the following four tests: Trail Making test (Strauss et al., 2006); Stroop Test (Golden, 1994); Digit Span backward/forward (WMS-R, Weschler, 1974); Hayling Test (Abusamra et al., 2007), and the fact that all of the participants were working or were able to work. Therefore, it may be the case that the difference between letter and category fluency in people with HIV only occurs in individuals who are cognitively impaired. To ratify these results, a longitudinal study may be considered. If factors potentially affecting fluency scores are controlled for (e.g., language/cognitive impairments, adherence to antiretroviral therapy; Schouten et al., 2014; Winston and Spudich, 2020), we would expect people with HIV to produce a similar number of words relative to healthy individuals over time (e.g., Crum-Cianflone et al., 2013). That is, they may produce fewer words in letter fluency than semantic fluency over time, but none of the scores would be different from that of healthy individuals (e.g., Vonk et al., 2020; for a longitudinal study including people with non-HIV dementia).

Regarding Aim 2, we found that people with HIV produced fewer words in letter fluency (*m*=23; SD=7) compared to animal (*m*=30; SD=10) and to unconstrained fluency (*m*=45; SD=16). This finding matches our predictions and can be explained by the fact that letter fluency is typically more difficult than semantic fluency (e.g., Vonk et al., 2020). This is because letter fluency poses more weight on the phonological output lexicon, as all the words selected need to start with a specific letter of the alphabet (e.g., “P”). This is not the case for animal fluency, where words need to be selected based on a criterion that is part of the semantic system (i.e., all words need to share the concept “animal”). Also, it is not the case for unconstrained fluency where, as we will argue in the following section, participants rely further on semantic criteria to produce words. Further on Aim 2, we found that people with HIV produced fewer words in animal fluency compared to unconstrained fluency. This was not expected. However, the current literature on this specific comparison is too small to provide any robust explanations (e.g., Abusamra et al., 2012). Therefore, we will explain these results by looking at the variables (i.e., cluster size, number of switches, and word properties) that were relevant to predict the total number of correct words.

As for Aim 3, we will first indicate that language functions and executive functions were involved in the performance of each of the fluency tasks. Interestingly, when we looked at the variance explained in each of the models, we saw that this ranged from 30 to 59%. We did not expect our models to explain 100% of the variance, as this would imply that the number of switches, cluster size, and word properties we entered in our analyses are the only factors that explain the total number of words for all the fluency tasks together, and for each fluency task separately. However, the amount of variance that was explained in each of the models can be considered to be high. In addition, most of the results could be cross-validated, by comparing the number of switches, cluster size, or word properties between individuals that performed within and below a normative sample in each of the tasks. The cross-validation is relevant because it supports our findings.

Specific to the argument of which types of language functions and executive functions are more engaged, we will argue that people with HIV rely on the phonological output lexicon, and not necessarily semantics, given that the variables that came up as relevant to predict the total number of words, namely, frequency and age of acquisition, have been most associated with the phonological output lexicon (e.g., Gilhooly and Watson, 1981; Cuetos et al., 2010; Whitworth et al., 2014). These findings match the current literature, as we will explain below, given that people with HIV have difficulties, albeit in our case limited, with lexical access and lexical retrieval (e.g., White et al., 1997; Millikin et al., 2004; Woods et al., 2004). Furthermore, we will argue that information updating and monitoring were most involved, as these types of executive functions are needed to renew the criteria used to search words and to keep track of the words produced and the task instructions (e.g., Miyake et al., 2000; Rofes et al., 2020).

Furthermore, we found that the number of switches was relevant to predict the total number of correct words, when considering a composite measure of the three tasks and when considering each task independently. This is understandable, because switches reflect two aspects of executive functions, namely, information updating and monitoring, which are necessary to adhere to the task instructions and to keep track of the words produced in any of the three tasks (Miyake et al., 2000; Rofes et al., 2020). Interestingly, the models indicated people with HIV that produced more words also produced more switches in animal and unconstrained fluency than in letter fluency (seven vs. six vs. three switches, respectively). This difference could reflect the pattern found in healthy individuals, whereby they produce less words in letter fluency compared to animal fluency (e.g., Vonk et al., 2020). The differences in number of switches, therefore, may indicate that producing more words also implies producing more words of different subcategories and, therefore, “switching more often” between subcategories (e.g., Mayr, 2002). An alternative explanation could be that people with HIV have difficulties with lexical access and lexical retrieval (e.g., White et al., 1997; Millikin et al., 2004; Woods et al., 2004). Therefore, they have more difficulty updating and monitoring information when a criterion that is found in the phonological output lexicon needs to be maintained (i.e., produce words that start with “P”), than when the criterion that needs to be maintained has a semantic nature (e.g., produce words of the category “animals”). In other words, the difficulties that people with HIV have at the lexical level make them produce less words in letter fluency (perhaps exacerbating the pattern that is already found in the general population, e.g., Vonk et al., 2020), and this is also seen with the number of switches.

Still, by looking at switches alone, we cannot explain why people with HIV may have more difficulty producing words in a letter fluency task than in an unconstrained fluency task. In that regard, studying cluster sizes and word properties may provide information to disentangle this issue. In our data set, cluster size showed as relevant to predict the total number of correct words in unconstrained fluency. This is in agreement with previous studies of unconstrained fluency in typically developing children and adults with right hemisphere damage (Beausoleil et al., 2003; Gonçalves et al., 2017). Furthermore, the relevance of cluster size over other variables in our data set stresses the argument that people with HIV do not have difficulties at the semantic level, while they may have them at the lexical level (e.g., White et al., 1997; Millikin et al., 2004; Woods et al., 2004). This is because clusters refer to groupings of successively generated words that belong to the same semantic family (e.g., Raskin et al., 1992; Troyer et al., 1997, 1998a,b; Troyer, 2000). Therefore, if the semantic system of people with HIV would have been damaged, cluster size would have probably not shown as relevant to predict the total number of words in unconstrained fluency tasks. If this argument holds, and people with HIV are more impaired at the lexical level but not at the semantic level, letter fluency should be more impaired than unconstrained fluency in people with HIV. This is also arguable from the perspective that unconstrained fluency poses more weight in the semantic system than in the phonological output lexicon (e.g., Beausoleil et al., 2003).

Another issue that may be resolved by looking at word properties of fluency tasks is explaining why individuals with HIV produced more words in unconstrained fluency than in animal fluency. Again, we will explain this issue from the perspective that people with HIV have difficulties with lexical access and lexical retrieval (e.g., White et al., 1997; Millikin et al., 2004; Woods et al., 2004). This is because, as we mentioned already, the total number of correct words for unconstrained fluency could be mostly explained with number of switches and mean cluster size. Alternatively, number of switches and frequency were the variables that explained the total number of correct words in animal fluency. The fact that frequency showed as relevant is important because this variable has been associated with the phonological and orthographic output lexica and because it has been argued not to be necessarily associated to semantics (e.g., Whitworth et al., 2014). This argument may seem a bit controversial, provided that animal fluency is typically argued as a task that requires semantic processes. Moreover, other studies have found that people with semantic dementia – a type of neurodegenerative disorder that is argued to predominantly impair the semantic system – show frequency effects (e.g., Lambon Ralph et al., 1998). However, the point we are trying to raise is not that animal fluency does not require semantic processes at all. As a matter of fact, we argue that it does require semantic processes when we discussed number of switches in the paragraphs above. What we are trying to argue here is that animal fluency may have further weight on lexical processes than unconstrained fluency. This should hold because, otherwise, in our data set, we would have found that variables that are more typically associated to the semantic system, such as imageability, concreteness, or familiarity, could explain the total number of words in animal fluency (e.g., Nickels and Howard, 1994, 1995; Guasch et al., 2016), while this was not the case. Furthermore, the fact that variables that relate to the phonological output lexicon can explain the results of animal fluency is not new. As in a previous study, we showed that age of acquisition could explain the total number of words in the animal fluency of people with AD (Rofes et al., 2020). This result should also not be surprising, given that age of acquisition and frequency are word properties that are argued to correlate (Ellis and Morrison, 1998).

This study has some limitations. First, the number of people with HIV that were included in the study (*N*=50) could be larger, even though the number is similar or larger to that of other studies of the same population (e.g., White et al., 1997; McCabe et al., 2002, 2008; Kambanaros et al., 2019; see cf. Millikin et al., 2004). At the same time, we included a data set of participants that shared some similarities, including the fact that none of them performed below the norm in a cognitive screening (MMSE ≥27/30, Butman et al., 2001) and, therefore, none of the participants were deemed cognitively impaired. Second, we included 10 different word properties and we considered word properties that have been related to different parts of the language system (i.e., semantic system, phonological output lexicon, and buffer) and that are commonly used in other studies and clinical work (e.g., Whitworth et al., 2014). However, given that the number of word properties that can be included is very large, it is possible that we did not include a word property that may be relevant for this specific population (e.g., Cutler, 1981). Third, the databases for age of acquisition, imageability, concreteness, valence, and arousal that we used were not developed with ratings from Spanish speakers from Argentina but from Spanish speakers from Spain. It would have been preferable to include databases that were developed for the Spanish that is spoken in Argentina. However, these databases are not yet available and, therefore, we considered that they could be used because the scores would correlate. To strengthen this point, we will mention that ratings for word properties associated with the phonological output lexicon and the semantic system (i.e., age of acquisition and imageability) correlate, even when the same words for typologically different languages are considered (e.g., Łuniewska et al., 2016; Rofes et al., 2018). Fourth, our participants and the normative sample we used were administered fluency tasks of 120s. However, other studies of fluency tasks, including studies running similar analysis to ours, have typically used 60s of data (e.g., Shao et al., 2014; Rofes et al., 2019, 2020). It is unclear whether having used more time could have biased the results or make our results less comparable to those of the current literature on word properties and fluency tasks. Still, the use of 120s is recommended when administering unconstrained fluency tasks (e.g., Beausoleil et al., 2003). Fifth, the results of the cross-validation for cluster size in unconstrained fluency did not reach significance. This means that we should interpret these results with caution and await replication in further studies, even though the algorithms that we used indicated that cluster size can predict the total number of words in unconstrained fluency. Sixth, many populations of people with HIV differ to normative sample in terms of lifestyle (e.g., alcohol, tobacco, and substance use) and other factors (e.g., depression, hypertension, impaired renal function, myocardial infraction, peripheral arterial disease, and trauma) that may affect cognitive results (Schouten et al., 2014; Winston and Spudich, 2020). Even though we did include a series of inclusion criteria, we could not control our participants for all possible factors. Also, the normative database we used does not report such data (Ferreres et al., 2007).

Finally, relative to future work, it may be relevant to assess the performance of people with HIV on other types of fluency tasks (e.g., actions, cities, supermarket items, and professions) that have been assessed in other populations (e.g., Monsch et al., 1992; Butler et al., 1993; Stokholm et al., 2013; Nogueira et al., 2016). This work may be useful to replicate the current findings and to assess whether people with HIV may have specific difficulties with different types of items (e.g., biological vs. non-biological; and within biological items: animals vs. fruits and vegetables) as this has been reported on people with other types of brain infections, such as people with Herpes Simplex Encephalitis (e.g., Gainotti, 2018). Another possible vector is to correlate the total number of correct words with scores from other tests that are typically attributed to measure specific language functions or other cognitive processes, such as executive functions (e.g., Henry et al., 2004; Shao et al., 2014; Whiteside et al., 2016). This latter exercise could be used, for example, to stress that people with HIV have language impairments in tasks that assess lexical processes or specific aspects of executive functions and that these difficulties can be seen in the same participants, when looking at specific properties of the words they produced in fluency tasks.

## Conclusion

People with HIV rely on language (phonological output lexicon, not necessarily semantics) and executive functioning (information updating and monitoring) to produce words in fluency tasks. The weight on these functions may vary, as indicated by the different importance of frequency, cluster size, and age of acquisition depending on the fluency task. These results await replication, for example, by correlating the total word number and other tests typically attributed to measure language and executive functions or by utilizing other types of fluency tasks (e.g., actions, cities, supermarket items, and professions).

## Data Availability Statement

The original contributions presented in the study are included in the article/Supplementary Material, further inquiries can be directed to the corresponding author.

## Ethics Statement

The studies involving human participants were reviewed and approved by the Ethics Committee of the Hospital Interzonal General de Agudos Eva Perón, Buenos Aires, Argentina. The patients/participants provided their written informed consent to participate in this study.

## Author Contributions

AR conceived the idea, developed the theory, retrieved the word properties from fluency tasks, and performed the statistical analyses. BS, LA, PC, VA, and RJ verified the analyses and supervised the findings. BS analyzed the clusters and switches. All authors contributed to the article and approved the submitted version.

## Funding

VA received funding from CONICET to conduct this research.

## Conflict of Interest

The authors declare that the research was conducted in the absence of any commercial or financial relationships that could be construed as a potential conflict of interest.

## Publisher’s Note

All claims expressed in this article are solely those of the authors and do not necessarily represent those of their affiliated organizations, or those of the publisher, the editors and the reviewers. Any product that may be evaluated in this article, or claim that may be made by its manufacturer, is not guaranteed or endorsed by the publisher.
